# Regional Cortical Thickness Predicts Top Cognitive Performance in the Elderly

**DOI:** 10.3389/fnagi.2021.751375

**Published:** 2021-11-04

**Authors:** Elena Nicole Dominguez, Shauna M. Stark, Yueqi Ren, Maria M. Corrada, Claudia H. Kawas, Craig E. L. Stark

**Affiliations:** ^1^Department of Neurobiology and Behavior, University of California, Irvine, Irvine, CA, United States; ^2^Mathematical, Computational and Systems Biology Graduate Program, University of California, Irvine, Irvine, CA, United States; ^3^Department of Neurology, University of California, Irvine, Irvine, CA, United States; ^4^Department of Epidemiology, University of California, Irvine, Irvine, CA, United States

**Keywords:** cortical thickness, cingulate cortex, top cognitive performer, successful aging, SuperAger, oldest-old

## Abstract

While aging is typically associated with cognitive decline, some individuals are able to diverge from the characteristic downward slope and maintain very high levels of cognitive performance. Prior studies have found that cortical thickness in the cingulate cortex, a region involved in information processing, memory, and attention, distinguish those with exceptional cognitive abilities when compared to their cognitively more typical elderly peers. Others major areas outside of the cingulate, such as the prefrontal cortex and insula, are also key in successful aging well into late age, suggesting that structural properties across a wide range of areas may better explain differences in cognitive abilities. Here, we aim to assess the role of regional cortical thickness, both in the cingulate and the whole brain, in modeling Top Cognitive Performance (TCP), measured by performance in the top 50th percentile of memory and executive function. Using data from National Alzheimer’s Coordinating Center and The 90 + Study, we examined healthy subjects aged 70–100 years old. We found that, while thickness in cingulate regions can model TCP status with some degree of accuracy, a whole-brain, network-level approach out-performed the localist, cingulate models. These findings suggests a need for more network-style approaches and furthers our understanding of neurobiological factors contributing to preserved cognition.

## Introduction

Advancements in health care and modern technology have led to an increase in life expectancy, such that by 2030, individuals 65 and older will outnumber those under the age of 18. Moreover, between 2000 and 2010, the United States saw a 30.2 and 29 percent increase in individuals aged 90–94 and 95+, respectively (US Census Bureau, 2010, 2018). With more individuals surviving until their ninth and tenth decades, many are interested in successful and healthy older aging. Contrary to those that may experience decline, some elderly individuals are able to remain disease free and maintain their cognitive abilities. Researchers have also identified subsets of individuals in their 70 and 80s that exhibit better-than-normal cognitive performance in comparison to their cognitively normal aged-matched peers. While various definitions of high-performance have been used, a common thread is well-preserved memory and executive functioning.

By examining morphological characteristics of the brain using structural neuroimaging, several studies have attempted to understand trajectories that are associated with avoiding decline and have started to elucidate what neurobiological factors contribute to preserved cognition throughout advanced aging. One representative group, known as SuperAgers, were distinguished based on their middle-age-like episodic memory, despite being in their eighties (Harrison et al., 2012; Gefen et al., 2015). Upon conducting a whole brain analysis, this high-performing group exhibited significantly greater cortical thickness in the cingulate cortex (Harrison et al., 2012). Note, however, that the modest sample sizes (*n* = 12 SuperAgers and *n* = 10 elderly controls) may have impacted their ability to reliably identify a broader range of regions. Following this finding, an *a priori* region-of-interest (ROI)-based analysis revealed that SuperAgers displayed greater cortical thickness in the posterior and caudal anterior cingulate cortex when compared to elderly controls (Gefen et al., 2015).

Studies of successful aging are not limited to the popularized SuperAger cohort and many have examined top performing individuals based on varying neuropsychological performance and tests. One commonality across studies has been highlighting the structural integrity of the cingulate cortex. Seventy-year-old successful agers, defined by high performance in episodic memory, working memory, and processing speed, displayed greater cortical thickness within the right anterior cingulate and prefrontal cortex (Harrison et al., 2018). Additionally, they had greater hippocampal volume and lower white matter hyperintensity volumes. Another study, examining optimal cognitive aging assessed by high performance in visuo-constructive abilities and visual reasoning, found that older individuals with high fluid abilities displayed greater cortical thickness in large areas of the cingulate cortex. Interestingly, they did not find this same relationship when comparing high vs. average performers in younger groups (Fjell et al., 2006).

Though the cingulate has proven to be important in successful aging, the structural integrity of other regions and networks have also been identified in preserved cognitions. For example, Sun et al. (2016) found that younger SuperAgers, aged 60–80, exhibited greater cortical thickness in the midcingulate, dorsomedial prefrontal cortex, angular gyrus, and superior frontal gyrus; all key regions in the default mode and salience networks. Whole brain analyses in high functioning individuals, aged 90 and older, revealed structural preservation in prefrontal and insular areas (Yang et al., 2016). Similarly, 70 + year old Successful Agers, distinguished by high memory scores, exhibited greater cortical thickness in the insula, midcingulate cortex, and the medial prefrontal cortex (Harrison et al., 2018). Thus, while the cingulate appears in each of these studies, several other regions have been implicated as well, suggesting a possible widespread network contributing to the resistance to cognitive decline.

It is important to note that our goal here is not to identify a set of specific cortical biomarkers of successful aging. Rather, the goal of the present study is a more generalized one. Here, we aim to understand how well cortical thickness can be used to model a behavioral outcome like successful aging, whether certain regions are disproportionately involved in this, and whether the cingulate cortex in particular is disproportionately involved. Thus, one hypothesis is that there is a set of specific regions, such as the cingulate regions, where thickness is able to predict cognitive status, while other regions have little or no predictive value (i.e., the cingulate is particularly informative when trying to model cognitive status). A second hypothesis is that the predictive power is distributed as a relatively smooth gradient across regions, with some more predictive than others, but no clear-cut differentiation between predictive and non-predictive regions. Finally, a third, “null” hypothesis is that all regions are equally predictive (or non-predictive) of cognitive status. Using structural and neuropsychological data from the National Alzheimer’s Coordinating center (NACC), we evaluated these hypotheses by examining the relationship between cortical thickness the brain and high cognitive performance in measures of episodic memory and executive function; two abilities that are otherwise known as hallmark domains of cognitive impairment and disease progression. We examined individuals aged 70–89, who demonstrated a combined performance at or above the top 50th percentile in both domains, deemed as Top Cognitive Performers (TCP) and we compared logistic regression performance using the cingulate ROIs relative to using the whole brain. To assess reliability of our models and the overall informativeness of individual regions, we performed Monte Carlo sampling of the population, creating logistic regression models for each sample. Finally, we examined the efficacy of these approaches as a function of age as by breaking them down by decade and including data from *The 90* + *Study.* Individuals in their 90s display a more marked and rapid decline than those in their 70s in cognitive domains such as memory, perceptual speed, knowledge, and fluency (Singer et al., 2003), making it valuable to understand how the informativeness of these metrics persists into very advanced stages of aging.

## Experimental Design and Methods

### The National Alzheimer’s Coordinating Center

#### Participants

**FIGURE 1:**
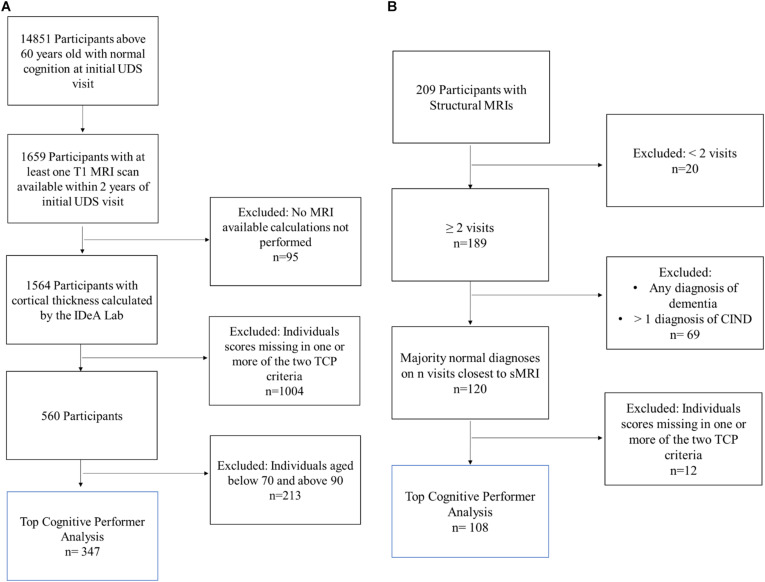
Inclusion flow chart for **(A)** NACC and **(B)** The 90 + Study Participants. Blue box reflects the participants included in the final analysis of top cognitive performers (TCP).

Three hundred and forty-seven individuals were selected from the larger NACC cohort (Figure 1A). NACC is a database of patient information collected from multiple Alzheimer disease centers funded by the National Institute on Aging (Beekly et al., 2004). For this analysis, participants were required to be seventy years old and above (70–89 years old) and have at least one T1 MRI scan available within 2 years of their initial UDS visit. Additionally, participants were required to have a NACC status indicating normal cognition and behavior (NORMCOG and NACCUDSD), as determined by a clinician or panel of clinicians based on neuropsychological test scores, CDR, Form B9 (Clinician Judgment of Symptoms), and center specific tests. Individuals who contained missing data in any of the criteria variables, described below, were excluded from the analyses.

#### Neuropsychological Criteria for Group Inclusion

Previous studies of successfully aging cohorts have used neuropsychological tests with specific criteria based either on performance being consistent with a younger population or with performance being atypically high for their age group. Following the latter, T were required to be in the top 50th percentile for both the Wechsler Memory Scale-revised Logical Memory IIA-Delayed Recall (WMS-R IIA) and Trails Making Test- Part B (Trails-B). The WMS-R IIA tests verbal and visual modalities and asks participants to recall units of a story after a 15 min delay (Wechsler, 1987). Trails-B engages executive function and processing speed by asking the participant to draw a line that connects an ordered progression of alternating letters and numbers (e.g., 1—A—2—B—3—C…) as quickly as possible (Tombaugh, 2004). All individuals that did not fit these criteria were classified as non-Top Cognitive Performers (non-TCP).

#### MRI Acquisition and Processing

Pre-calculated regional cortical thickness data for NACC MRIs were provided by the IDeA Lab at University of California, Davis. T1-weighted structural MRI (sMRI) scans were obtained from multiple centers using 3.0 and 1.5 Tesla scanners (GE, Siemens, and Phillips). sMRI data from the date closest to the initial UDS visit were processed based on the Advanced Normalization Tools (ANTs) toolkit and thickness pipeline (Das et al., 2009). Modifications to that pipeline for improving GM/WM segmentation used to generate the numbers in NACC are described by Fletcher et al. (2012).

### The 90 + Study

#### Participants

One hundred and eight individuals from the larger *The 90* + *Study* cohort were included (Figure 1B). The 90 + Study, established in 2003, is an ongoing longitudinal investigation of aging and dementia in individuals aged 90 and above, consisting of the survivors of the Leisure World Cohort Study (Kawas, 2008). Participants were selected based on the availability of a sMRI, two or more neuropsychological visits, and a cognitively normal diagnosis at a majority of their visits (i.e., 2 out of 3 visits or 3 out of 4 visits). Cognitively normal was determined by *The 90* + *Study* and refers to a primary diagnosis, determined by neurological examiners, where an individual is deemed as normal, absent of impairment in any cognitive domains, and able to complete Instrumental activities of daily living (IADL). Individuals who contained missing data in any of the criteria variables were excluded from the analyses.

#### Neuropsychological Criteria for Group Inclusion

While participants in *The 90* + *Study* are visited every 6 months by researchers who perform neuropsychological tests, the number of visits for each individual at the time of these analyses varied from 1visit to 23. Thus, based on the available data, median cognitive scores from up to four visits closest to sMRI scan date were chosen as a more robust measure of cognition that would account possible variance in individual session performance. Following NACC TCP criteria, The 90 + TCP individuals were required to perform at or above the top 50th percentile for their age group on the long-delay recognition portion of the California Verbal Learning Test—short form (CVLT) and at or above the top 50th percentile on completion time for their age group in the Trails-B. All other individuals that did not fit these criteria were classified as non-Top Cognitive Performers (non-TCP).

#### MRI Acquisition and Processing

**FIGURE 2:**
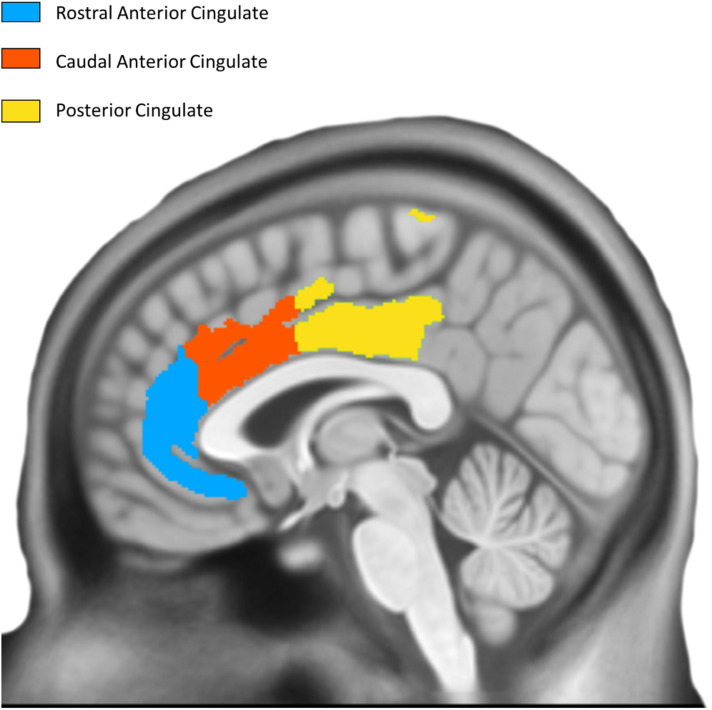
Desikan Killiany Tourville Atlas Three bilateral *a priori* cingulate regions derived from DKT atlas; left hemisphere is shown.

T1-weighted structural MRI scans were collected on a 3.0 Tesla GE Discovery MR750w scanner (1 mm isotropic resolution, TE = 3 ms, TR = 7.2 ms, flip angle = 11°). Images were processed using Mindboggle (Klein et al., 2017), which performs atlas registration to the Desikan-Killiany-Tourville (DKT) atlas (Desikan et al., 2006) and cortical thickness estimation using Advanced Normalization Tools (ANTs; its additional FreeSurfer estimates were not used here). ANTs calculates cortical thickness by measuring the distance between gray/white matter boundaries and gray/CSF boundaries by quantifying the amount of registration needed to bring these surfaces together. Thickness was calculated in the original native subject space before being transformed into MNI space. Using DKT regions of interest as masks, we computed the average cortical thickness within each ROI. To reduce edge effects that will be present in these masks (thickness is computed in the cortical sheet and the ROIs will cover voxels not in a particular subject’s sheet), thickness maps were clipped at 1mm and the average computed across all resulting non-zero voxels. The average cortical thickness of three bilateral cingulate regions from the DKT atlas (posterior cingulate cortex, caudal anterior cingulate cortex, rostral anterior cingulate cortex) was examined as *a priori* regions based on their previously shown involvement in successful aging (Figure 2).

### Statistical Analysis of MRI Study Participants and Cortical Thickness

For both datasets, statistical analyses were performed using SAS and both the Statsmodels^1^ and skikit-learn^2^ libraries in Python. To evaluate the influence of cortical thickness on top cognitive performance (TCP), two logistic regression model were used to model TCP status as a function of regions of interest as follows: (1) 6 bilateral *a priori* cingulate ROIs (rostral anterior, caudal anterior, and posterior segments), and (2) a forward-selection model with 62 whole brain cortical ROIs (cutoff *p*-value for the F statistic, *p* = 0.25). Receiving Operating Characteristic (ROC) curves were created to assess the accuracy of TCP status as a diagnostic marker. Additionally, unpaired *t*-tests were used to evaluate differences in continuous variables (age, education-NACC, and neuropsychological performance) and Fisher’s exact test to evaluate gender distribution, across the two subject groups.

## Results

### Demographics and Neuropsychological Performance at Baseline

NACC analyses used data from 11 Alzheimer’s Disease Research Centers (ADRCs) for UDS visits conducted between September 2005 and December 2020. The average time between initial neuropsychological visit and MRI was 133.3 (189.6) and 138.7 (185.1) days for non-TCP and TCP, respectively. The 347 NACC participants had an average of 15 years of education and were 60.81% female (Table 1). TCP and Non-TCP groups did not differ in age in either the 70 [*t*(24) = 0.71, *p* = 0.48] or 80 year old subgroups [*t*(1) = 0.98, *p* = 0.33]. They did, however, differ in education [*t*(242) = 5.02, *p* < 0.0001] and gender distribution (Fisher’s exact *p* = 0.04) in the 70 year-olds and education [*t*(101) = 2.72, *p* = 0.01] in the 80 year-olds.

**TABLE 1:** NACC demographics.

	**All**	**70 year old’s**	**TCP**	**Non-TCP**	***T*-test/Fisher’s exact test**	**80 Year Old’s**	**TCP**	**Non-TCP**	***T*-test/Fisher’s Exact Test**
n	347	244	83	161		103	22	81	
Age (SD)	75.94 (4.95)	74.25 (2.72)	74.07 (2.68)	74.34 (2.75)	0.476	83.31 (2.67)	82.82 (2.56)	83.44 (2.70)	0.331
Female (%)	211 (60.81%)	150 (61%)	59 (71%)	91 (56%)	**0.037**	61 (59.22%)	13 (59.09%)	48 (59.26%)	>0.999
Education (SD)	15.00 (3.38)	15.07 (3.35)	16.49 (2.37)	14.33 (3.54)	** < 0.0001**	14.82 (3.47)	16.55 (2.54)	14.35 (3.55)	**0.008**

*NACC sample subject demographic information with T-test and Fisher’s Exact Test comparisons. *p* value < 0.05.*

The 108 90 + Study participants were 63.89% female and 47% had a college education (Table 2). TCP and Non-TCP did not differ in age [*t*(106) = 0.58, *p* = 0.57], gender distribution (Fisher’s exact *p* = > 0.999), or education level at a baseline visit [*X*^2^(2, *n* = 108) = 1.88, *p* = 0.39].

**TABLE 2:** The 90 + study demographics.

	**All**	**TCP**	**Non-TCP**	***T*-test/Fisher’s exact test or chi square test**
n	108	35	73	
Age (SD)	93.85 (2.60)	94.06 (2.60)	93.75 (2.62)	0.565
Female (%)	69 (63.89)	22 (62.86)	47 (64.38)	>0.999
Education (SD) High-school graduate or less (%) Some college to college graduate (%) Some graduate school or higher (%)	15 (13.89) 47 (43.52) 46 (42.59)	3 (8.57) 18 (51.43) 14 (40)	12 (16.44) 29 (39.73) 32 (43.84)	0.391

*The 90 + Study sample subject demographic information with T-Test, Fisher’s Exact Test, and Chi square test comparisons.*

### Cortical Thickness in the National Alzheimer’s Coordinating Center Sample: *A priori* Cingulate Regions

When considered in isolation, logistic regressions modeling TCP status based on the *a priori* cingulate regions’ thickness (Table 3) failed to robustly model TCP status. When examining the full NACC 70–89 sample, no cingulate ROI could reliably model TCP status (*p*’s > 0.1, uncorrected for multiple comparisons). When restricted to only those in their 70s, the right caudal anterior (*p* = 0.04, uncorrected), and rostral anterior (*p* = 0.01, uncorrected) cingulate showed some predictive power, but this was not the case in the NACC participants in their 80s (*p* = 0.15 and *p* = 0.29, respectively).

**TABLE 3:** Fitted logistic regression models.

**Age Group**	**ROI**	**Fitted Model**
70–89	Left Caudal Anterior Cingulate, Left Posterior Cingulate, Left Rostral Anterior Cingulate, Right Caudal Anterior Cingulate, Right Posterior Cingulate, Right Rostral Anterior Cingulate	Logit(TCP) = −4.13 + 0.48 x_*LCaudal Anterior Cingulate*_ + 0.48 x_*LPosterior Cingulate*_ + 0.28 x_*LRostral Anterior Cingulate*_ + 0.43 x_*R Caudal Anterior Cingulate*_ + 0.33 x_*RPosterior Cingulate*_ - 0.61 x_*RRostral Anterior Cingulate*_
	**Left Caudal Anterior Cingulate**, Left Caudal Middle Frontal, Left Entorhinal, Left Medial Orbitofrontal, Left Paracentral, Right Cuneus, Right Superior Frontal	Logit(TCP) = −2.36 + 0.66 x_*LCaudal Anterior Cingulate*_ + 1.72 x_*LCaudal Middle Frontal*_ - 0.62 x_*LEntorhinal*_ + 1.28 x_*LMedial Orbitofrontal*_ + 1.37 x_*L Paracentral*_ − 1.00 x_*RCuneus*_ − 2.23 x_*RSuperior Frontal*_
70s	Left Caudal Anterior Cingulate, Left Posterior Cingulate, Left Rostral Anterior Cingulate, Right Caudal Anterior Cingulate, Right Posterior Cingulate, Right Rostral Anterior Cingulate	Logit(TCP) = −3.95 + 0.73 x_*LCaudal Anterior Cingulate*_ + 0.28 x_*LPosterior Cingulate*_ + 0.51 x_*LRostral Anterior Cingulate*_ + 1.05 x_*RCaudal Anterior Cingulate*_ + 0.10 x_*RPosterior Cingulate*_ − 1.28 x_*RRostral Anterior Cingulate*_
	Left Entorhinal, Left Inferior Temporal, Left Paracentral, **Left Rostral Anterior Cingulate, Right Caudal Anterior Cingulate**, Right Lingual, **Right Rostral Anterior Cingulate**	Logit(TCP) = −4.19 - 0.64 x_*LEntorhinal*_ + 1.01 x_*LInferior Temporal*_ + 1.62 x_*LParacentral*_ + 1.19 x_*LRostral Anterior Cingulate*_+ 1.33 x_*RCaudal Anterior Cingulate*_ − 1.50 x_*RLingual*_ − 1.39 x_*RRostral Anterior Cingulate*_
80s	Left Caudal Anterior Cingulate, Left Posterior Cingulate, Left Rostral Anterior Cingulate, Right Caudal Anterior Cingulate, Right Posterior Cingulate, Right Rostral Anterior Cingulate	Logit(TCP) = −5.37 − 0.30 x_*LCaudal Anterior Cingulate*_ + 1.91 x_*LPosterior Cingulate*_ − 0.24x_*LRostral Anterior Cingulate*_ − 1.06 x_*RCaudal Anterior Cingulate*_ + 0.74 x_*RPosterior Cingulate*_ + 0.77 x_*RRostral Anterior Cingulate*_
	Left Pericalcarine, Left Postcentral, Left Superior Temporal, Left Supramarginal, Right Isthmus Cingulate, Right Parahippocampal, Right Superior Parietal	Logit(TCP) = −9.94 + 3.49 x_*LPericalcarine*_ + 9.14 x_*LPostcentral*_ − 5.33 x_*LSuperior Temporal*_ + 3.95 x_*LSupramarginal*_+ 5.76 x_*RIsthmus Cingulate*_− 3.72 x_*RParahippocampal*_ − 7.79 x_*RSuperior Parietal*_
90s	Left Caudal Anterior Cingulate, Left Posterior Cingulate, Left Rostral Anterior Cingulate, Right Caudal Anterior Cingulate, Right Posterior Cingulate, Right Rostral Anterior Cingulate	Logit(TCP) = −2.67 + 1.51 x_*LCaudal Anterior Cingulate*_ + 0.55 x_*LPosterior Cingulate*_ − 0.11x_*LRostral Anterior Cingulate*_ − 1.28 x_*RCaudal Anterior Cingulate*_ + 0.71 x_*RPosterior Cingulate*_ − 0.43 x_*RRostral Anterior Cingulate*_
	Left Isthmus Cingulate, Left Lateral Orbitofrontal, Left Pars Opercularis, Left Transverse Temporal, **Right Caudal Anterior Cingulate**, Right Medial Orbitofrontal, Right Insula	Logit(TCP) = −4.21 + 2.31 x_*LIsthmus Cingulate*_ + 5.41 x_*L Lateral Orbitofrontal*_ − 6.94 x_*LPars Opercularis*_+ 2.25 x_*LTransverse Temporal*_ − 2.63 x_*RCaudal Anterior Cingulate*_− 2.77 x_*RMedial Orbitofrontal*_ + 2.33 x_*RInsula*_

*First row for each respective age group (70–89, 70s, 80s, and 90s) represents logistic regression models ran with a priori cingulate ROIs as predictors. Second row for each respective age group represents forward selection logistic regression models ran with all 62 cortical ROIs and each region selected; cingulate regions are bolded for reference*.

**FIGURE 3:**
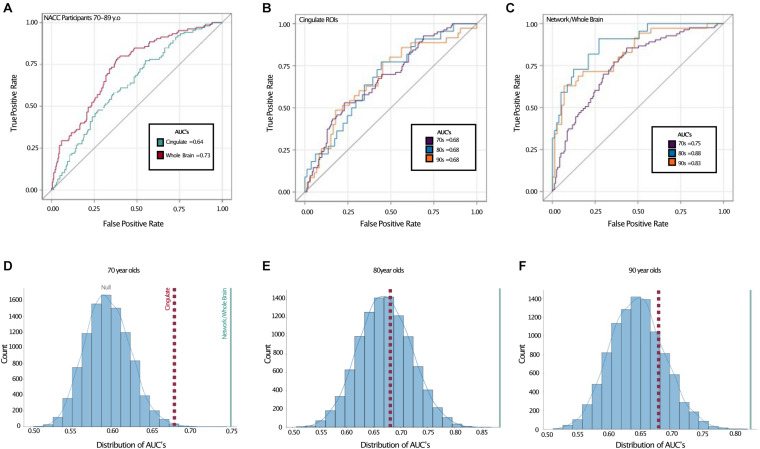
Receiver operating characteristic curves show better TCP predictive performance in whole brain model. Top: ROC curves with area under the curves (AUC) displayed for **(A)** entire NACC sample (ages 70–89), **(B)** *a priori* cingulate regions in all age groups, and **(C)** network/whole-brain forward-selected ROIs across all age groups. Bottom: Permutation analyses where the labeling of TCP vs. non-TCP was shuffled 10,000 times in **(D)** NACC 70 year olds, **(E)** NACC 80 year olds, and **(F)** The 90 + Study 90 year olds. Red dotted lines represent AUC’s for *a priori* cingulate ROIS reflected in **(B)** and blue solid lines represent AUC for network/whole-brain forward-selected ROIs reflected in **(C)**. AUC: Area under the curve, 70s: NACC 70 year olds, 80s: NACC 80 year olds, 90s: The 90 + Study 90 year olds.

We next turned to receiver-operating characteristic (ROC) analysis, using a logistic multiple regression based on all six cingulate ROIs modeling TCP status. Here, we used the area under the curve (AUC) to quantify performance and assess the sensitivity and specificity of the model. Using this, the cingulate regions yielded an estimated AUC of 0.64 across the full age range in NACC (Figure 3A). While modest, this AUC was reliably better than chance. Given the combination of the biased base-rate of TCP status and the multiple predictors used (and potential for overfitting), a null value of 0.5 for AUC cannot be assumed. To assess the null and estimate the true alpha, we conducted a permutation analysis, randomly shuffling the TCP/non-TCP labels 10,000 times and running the same logistic regression and AUC estimation to empirically derive an alpha using the same data and the same proportion of TCP status labels. We found the alpha to be ∼0.009, indicating the odds that a large or larger AUC would be generated by chance (Figure 3D, blue line).

### Cortical Thickness in the National Alzheimer’s Coordinating Center Sample: Whole-Brain

To determine whether the cingulate ROIs represented the ideal or near-ideal set of regions for this approach, we next performed a whole-brain forward-selection logistic regression (i.e., using all 62 cortical ROIs). This analysis selected the left caudal anterior cingulate, left caudal middle frontal, left entorhinal, left medial orbitofrontal, left paracentral, right cuneus, and right superior frontal regions with a resulting AUC of 0.74 and a permutation-derived alpha of *p* < 0.0001. Thus, while one of the cingulate regions was present in this model, the optimal model drew upon regions throughout the brain.

We should note that this is not the result of any global difference in cortical thickness across TCP groups. Estimates of average whole-brain cortical thickness were calculated for each individual by weighted averaging of the thickness from all 62 regions (weighted by region volume). Unpaired *t*-tests showed no difference in average whole-brain cortical thickness for the whole cohort or for those in their 70s or 80s separately (all *t*’s < 0.8, all *p*’s > 0.4).

### Cortical Thickness Across Age Groups

We next turned to the question of whether our ability to model TCP status was affected by age. To do so, we shifted from thickness values provided by NACC to thickness values derived from ANTs directly as we wanted to include data from The 90 + Study as well to give a broader age range (note, we found that overall, the estimates provided by NACC yielded slightly higher AUCs than those provided by ANTs.) Here, we found that when restricting ourselves to the *a priori* cingulate regions, all three age groups yielded virtually identical ROC curves and 0.68 AUC values (Figure 3B). As with the combined data, however, shifting to a whole brain analysis improved performance considerably. The AUCs rose to 0.75 in the cohort in their 70’s, 0.88 in those in their 80’s, and 0.83 in the 90 + (alpha < 0.0001 in all). ROC contrast estimations comparing AUC’s for cingulate vs. forward-selected ROIs revealed a significant difference across all age groups, suggesting a better fit by regional cortical thickness (all *p* < 0.02).

### Role of Age, Sex, and Education Covariates

Our primary question here was whether cortical thickness could be used to model TCP status. As such, we excluded typical covariates such as age, sex and education that might otherwise predict TCP status and therefore inflate our AUC values. To determine their predictive value beyond cortical thickness, we repeated each of these logistic regression models including these factors. There was a slight increase in AUC’s across all age groups when age, sex, and education were added to the model (Table 4). The 70 year old group showed the largest improvement, moving from 0.75 to 0.8, while the 80 year old group improved from 0.88 to 0.89 and the 90 year old group from 0.83 to 0.85.

**TABLE 4:** Effects of age, sex, and education on AUC values.

**Age group**	**Forward selected ROIs**	**Forward selected ROIs + sex**	**Forward selected ROIs + education**	**Forward selected ROIs + sex, education**	**Forward selected ROIs + age, sex, and education**
70–89	0.73	0.74	0.77	0.79	0.79
70s	0.75	0.76	0.78	0.80	0.80
80s	0.88	0.89	0.89	0.90	0.89
90s	0.83	0.83	0.84	0.84	0.85

*To assess predictive ability beyond cortical thickness, age, sex, and education were added to logistic regression model.*

### Reliability of Selected Regions

Finally, we turned to the question of the consistency of the generated models. Informally, Table 3 shows that there is some degree of consistency across models, but that there is significant deviation in the regions chosen as well. These data lead to the hypothesis considered at the outset that, rather than a specific set of ROIs carrying far more predictive value than others, that all ROIs capture some amount of this variance. In this framework, which ROIs are selected in the model might depend, to a large degree, on the specific sample of brains used rather than purely any prior probability of the predictive value of a given region.

**FIGURE 4:**
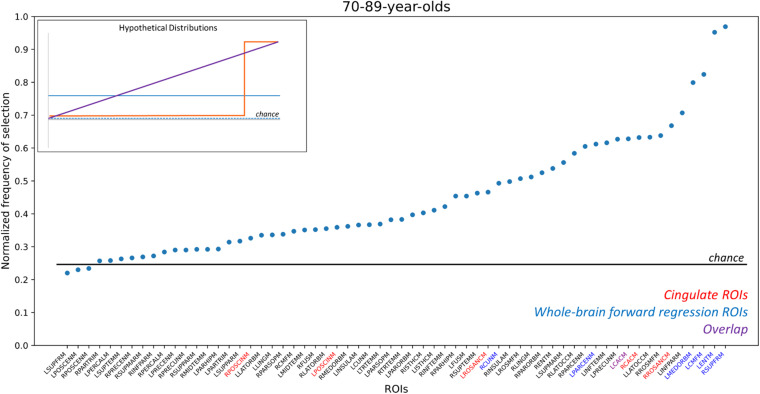
Frequency of ROI selection in bootstrapping analysis. Random samplings of subjects matching our existing TCP rates were repeatedly drawn and analyzed using the same logistic forward regression to determine how often each ROI was selected by the model. ROIs here are sorted by their frequency of being selected, which was normalized by the number of iterations (*n* = 1,000) to scale from 0 to 1, and ROI names are color-coded by whether they are part of the cingulate (*red*), were in the original whole-brain model (*blue*), or both (*purple*). The horizontal line reflects the chance frequency of selection. ROI names are based on NACC labels. RPOSCINM, Right Posterior Cingulate; LPOSCINM, Left Posterior Cingulate; LROSANCM, Left Rostral Anterior Cingulate; RCUNM, Right Cuneus; LPARCENM, Left Paracentral; LCACM, Left Caudal Anterior Cingulate; RCACM, Right Caudal Anterior Cingulate; RROSANCM, Right Rostral Anterior Cingulate; LMEDORBM, Left Medial Orbital; LCMFM, Left Caudal Middle Frontal; LENTM, Left Entorhinal; RSUPFRM; Right Superior Frontal. For full list, please refer to NACC’s Imaging Data Researcher’s data dictionary: https://files.alz.washington.edu/documentation/rdd-imaging.pdf. Inset figure: Hypothetical distributions that would arise from different underlying models: (*orange*) distribution that would result if only a small subset of regions were highly predictive; (*purple*) distribution that would result if an even gradient of predictive ability existed across regions; (*blue, dashed*) distribution that would result if no regions’ cortical thickness could model TCP status; (*blue, solid*) distribution that would result if all regions had some predictive power but there was no differentiation across regions.

Here, we sought to determine the distribution of the predictive value of each ROI across samplings. To do so, we performed a non-parametric bootstrapping analysis that drew 1,000 samples from our 70 to 89 NACC population. Each sample drew the same number of TCP and non-TCP individuals as our final dataset, drawing samples with replacement to arrive at an estimate of the samples one might have outside of our particular population (Wu and Jia, 2013). Figure 4 shows the resulting distribution of how often each region was selected in the forward-selection logistic along with an inset depicting several possible models. This enables us to determine whether a subset of regions is selected more often than others (e.g., inset, orange line), all regions are equally likely to be selected (inset, blue solid or dashed lines), or if some in between gradient of predictive value for regions exists (inset, purple line). Results showed a curvilinear distribution that highlighted the relative importance of some regions, but also demonstrated the broad predictive value across the whole brain. In particular, two regions (left entorhinal and right superior frontal) were selected in almost every iteration with two more (left caudal middle frontal and left medial orbitofrontal) selected over 75% of the time. Notably these four ROIs were also included in our initial forward-selection model. Moving down in frequency of selection, 11 ROIs were selected ∼60–70% of the time. This group included three of the *a priori* cingulate ROIs. Note, all of the 62 ROIs were selected at least 22% of the time, although some number of these are at rates expected by chance (determined by 1,000 random shufflings of the TCP/non-TCP labels and repeating the entire process to determine the base rate of region selection). When this analysis was repeated in the 70 year-olds separately, the two of the top four ROIs in the whole group were again in the top 4 here, but the overall distribution was quite linear (Supplementary Figure 1A). When repeated in just the 80-year olds, the distribution was again non-linear, but no region was included more than 62% of the iterations and none of the original top four ROIs were present in the top four in this subset (Supplementary Figure 1B).

## Discussion

The present study aimed to: (1) assess if the cingulate as a localized *a priori* network sufficiently models successful aging, (2) observe if such relationships between cortical thickness and TCP persists in rising age groups, and (3) assess the reliability of various selected networks in the brain in modeling TCP. We were particularly interested in the cingulate cortex based on it recurring role in successful aging literature, as well its role in cognition; including information processing, memory, emotional processing, task engagement, and attention (Vaidya et al., 2007; Pearson et al., 2011; Stanislav et al., 2013). Here, we were able to replicate the finding that the thickness of cingulate cortex can be used to some degree to model TCP status and that this ability was similar across 70s, 80s, and 90 + cohorts (Figure 3B). However, we also found that far stronger models could be made when extending the scope of the analysis to the whole brain. Our AUCs from the ROC analyses revealed that, across all age groups, forward selected ROIs from the logistic regression outperformed *a priori* cingulate regions in modeling TCP status. Furthermore, the regions selected by logistic regressions, either on the complete dataset (Table 4) or via random sub-sampling of our data (Figure 4) often had representation of the cingulate (typically caudal anterior cingulate), but also included representation across the brain.

Thus, while our results continue to implicate structural characteristics of the cingulate cortex in successfully aging individuals, these results suggest that global-style networks, rather than literature driven localized areas, may be better at modeling preserved cognition in the elderly. This is not to suggest a new subset of regions as a model for studying successful aging, but rather to propose examining a more data-driven set of ROIs as a robust approach in modeling superior cognition in memory and executive function.

Similar relationships can be found in other modes of imaging. Seventy-year-old “supernormals,” defined by stringent criteria based on 5-year maintenance of episodic memory and executive functioning, displayed stronger functional connectivity between anterior cingulate and the hippocampus, middle cingulate, posterior cingulate, among other regions when compared to healthy elderly controls and those with mild cognitive impairment (MCI) (Lin et al., 2017). More importantly, these researchers identified a functional “Supernormal map,” consisting of the right fusiform gyrus, right middle frontal gyrus, right anterior cingulate cortex, left middle temporal gyrus, left precentral gyrus, and left orbitofrontal cortex, which successfully predicted a 1-year change in global cognition and correlated to Alzheimer’s pathology (Wang et al., 2019). Similar to possible cortical signatures of successful aging, these findings all suggests a pattern of widespread brain regions that may reflect the neurobiological underpinnings that result in preserved cognition.

This widespread pattern is perhaps best illustrated in Figure 4 where we aggregated across many random resamplings of our 70–89 year-old population to determine how frequently different regions were included in our logistic model. Under the null hypothesis of all regions being equally uninformative of TCP status (inset, blue dashed), we would have observed a flat distribution with all regions being included ∼25% of the time. Under a localistic hypothesis in which some subset of regions are informative of TCP status while others are not (inset, orange line), we would have observed a step-function distribution where most regions were uninformative and highly unlikely to be included in the model while others were highly informative and almost always included in the model. Our results were not consistent with either of those hypotheses, instead supporting the view that while cortical thickness is informative of TCP status and while individual regions do vary in their predictive value, there is no specific subset of regions that are the key regions we should use. Instead, the results suggest that many, if not all regions carry the ability to inform modeling of TCP status. Thus, specific set of regions one isolates in a given analysis from a given sample of scans will vary to some degree from what one would arrive at with a different set of scans. However, these results do not arise from simple Type II error as shown by the permutation analyses in Figures 3D–F and by the distribution shown in Figure 4. Rather, if all regions contain variance that is informative of TCP status to some degree, we would expect that noise and the randomness associated with a particular population (that which we attempted to model in Figure 4) will lead to a somewhat different subset of ROIs being chosen in any particular forward selection model, consistent with what we observed in Table 4. Therefore, when approaching the problem of modeling TCP status from regional cortical thickness, we must view this as a “brain-wide” problem rather than a “localistic” problem. Rather than approaching a problem such as the relationship between a biomarker like cortical thickness and a behavioral outcome such as TCP status by searching for a critical region or small set of regions, a richer understanding of this relationship might be had by taking a more “distributed” or network-based approach.

While discussing this network-level view of relating regional thickness to TCP status, we should note that a number of the beta coefficients in our models (Table 3) are negative. These negative coefficients should not be interpreted as demonstrating a thinner cortex in these regions in TCP individuals. For example, in our 70–89 group, while approximately half of the coefficients in Table 3 are negative in both the cingulate and the whole-brain analyses, TCP individuals are numerically thicker in all these regions (see also Figure 3). The negative coefficients are merely the byproduct of this multiple regression approach.

Finally, we should note that the group analyses in NACC revealed significant differences in sex and education. TCP subjects in their 70s tended to have a higher education and female distribution, while those in their 80s tended to only be more highly educated. All AUCs increased when both sex and education were added into the model, but the gains in AUC appeared quite modest. This is not to say that sex and education are not informative of cognitive status. When examining a cohort of SuperAgers from the Personality and Total Health Through Life (PATH) study, researchers found that SuperAging was both more prevalent in woman and associated to education (Maccora et al., 2021). It is possible that the significant differences in demographics are attributed to TCP group inclusion, which is reflected higher scores in both memory and executive function. Previous studies examining the role of age, sex, and education in elderly cognition revealed that (1) individuals with higher levels of education performed better on cognitive tests and (2) women performed better than men on verbal memory tasks (van Hooren et al., 2007). Additionally, despite there being no group differences in the oldest-old TCP, The 90 + Study previously showed that higher education is associated with lower prevalence rates of dementia in women (Corrada et al., 2008). For example, if education and sex alone are used to model TCP status in the 70–89 group, performance is at least as good as the *a priori* cingulate-only models (AUC = 0.7 vs. the cingulate’s AUC of 0.64). However, it is not the case that thickness is merely a very expensive way of determining age and education, as in other groups, performance is far worse (e.g., 90 + Age + Education AUC = 0.52 vs. cingulate AUC = 0.68 or whole-brain AUC = 0.83). Therefore, it is clear that cortical thickness, while potentially correlated with these other factors, can be used to model TCP status irrespective of them.

### Limitations

While participants were required to be diagnosed as cognitively normal, and thus determined to be free from MCI or dementia, it is possible that we are capturing some non-TCP individuals who are pre-clinical, defined here as asymptomatic participants with evidence of AD pathology or individuals who display cognitive symptoms that do not meet clinical criteria for MCI. AD-related lesions accumulate in the brain years before cognitive deficits (Morris and Price, 2001; Rowe et al., 2007), with longitudinal studies showing amyloid deposition measured by PET 15 years before symptom onset (Bateman et al., 2012). Thus, it is possible that the effects observed can be attributed to other aging biomarkers not captured on structural MRI, especially given the wide age range. Future studies examining such biomarkers will be informative for better understanding differences in the available data.

We should also point out that the behavioral measures chosen for these analyses were based on previous successful aging studies, which typically use a test of delayed recall [usually CVLT or Rey Auditory Verbal Learning Test (RAVLT)] and executive function (usually Trails B). The WMS-R IIA and CVLT were chosen as tests of delayed recall to mirror this standard as closely as possible, limited by what data is available in each dataset. There are some key differences between these tests of delay recall potentially influencing the differences found between cohorts. The WMS-R IIA requires participants to recall units of a provided story while the CVLT requires participants to recall words from a list. While a narrative will help memory and can be used in both cases, any such narrative must be constructed by the participant in the case of the CVLT, leading to potentially more contamination by executive function in a word list task like the CVLT (Tremont et al., 2000). While these tests are not identical in nature, both are measures of verbal memory and tap into strategic organization of the information to help memory and we find it more likely that the differences observed between cohorts are better explained by additional complex changes the aging brain goes through that may change the importance of structural characteristics of certain brain regions throughout the lifespan, such as amyloid deposition or vascular changes. It is important to note that differences were also observed within NACC throughout age groups, thus making it less likely that differences are attributed test type.

In addition to the relatively modest sample sizes (particularly in the 80− and 90-year old subgroups), it is important to note the role of volunteer and selection biases these analyses common to many aging studies and potentially all neuroimaging studies. People who are able and willing to participate in imaging tend to be healthier and meet *a priori* selection criteria. One large study examining the nature of volunteer and selection biases found that those who were more likely to participate in studies were also more likely to be cognitively healthy, well-educated, and male compared to their counterparts who were not interested in participating (Ganguli et al., 2015). Inclusion criteria and recruitment, amongst other factors, have led to a more heterogenous population in NACC participants, which tend to be mostly Caucasian and of both high socioeconomic and education status. Given that participants from The 90 + Study are largely survivors of *Leisure World Cohort Study* and recruited from a retirement community in Laguna Woods, California, it is certainly possible that participants are not fully representative of the population. As reported by Melikyan et al. (2019), compared with the oldest-old population in the United States (He and Muenchrath, 2011), the cognitively normal sample in *The 90* + *Study* has a higher proportion of Caucasians (98.5% vs. 88%) and a higher percentage of individuals with more than a high school education (78% vs. 28%). Previous research has shown that differences in sex and education may account for cognitive test performance (Hall et al., 2007; van Hooren et al., 2007), and cortical thickness (Seo et al., 2011; Habeck et al., 2020; Steffener, 2021). As reported in Tables 1, 2, the overall TCP NACC sample was 61% female with had an average of 15 years of education, while The 90 + Study sample was 64% female and 46% college educated and above. It is possible that higher education or larger female distributions may be influencing external validity by: (1) introducing a moderation relationship between key demographics, test performance, and cortical thickness that we would not otherwise see in the general public or (2) significantly influencing the distribution of TCP (approximately 32 and 30% for current NACC and The 90 + Study analyses, respectively) that is not representative of all elderly individuals. It is also important to note that, given these potential biases and the fact that percentiles for TCP group inclusion were determined based on a very select subset of each of these cohorts (blue boxes reflected in Figure 1), inclusion in the top 50th percentile for each of our cognitive domains may not reflect TCP in the general public. Finally, we should note that the present study cannot identify specific mechanisms that are associated with these differences in cortical thickness.

## Data Availability Statement

Publicly available datasets were analyzed in this study. National Alzheimer’s Coordinating Center data can be requested here: https://naccdata.org/requesting-data/submit-data-request.

## Ethics Statement

The studies involving human participants were reviewed and approved by the University of California, Institutional Review Board for the 90 + Study. The studies involving data from the NACC were reviewed and approved by their respective IRBs. The patients/participants provided their written informed consent to participate in this study.

## Author Contributions

CK, CS, and ED conceived of the project. ED, YR, and CS performed data analyses. All authors contributed to writing and editing the manuscript.

## Conflict of Interest

The authors declare that the research was conducted in the absence of any commercial or financial relationships that could be construed as a potential conflict of interest.

## Publisher’s Note

All claims expressed in this article are solely those of the authors and do not necessarily represent those of their affiliated organizations, or those of the publisher, the editors and the reviewers. Any product that may be evaluated in this article, or claim that may be made by its manufacturer, is not guaranteed or endorsed by the publisher.

## Footnotes

1
https://www.statsmodels.org/

2
https://scikit-learn.org/
