# Supplementary material for: Regional Cortical Thickness Predicts Top Cognitive Performance in the Elderly
Source: Front Aging Neurosci. 2021 Nov 4;13:751375. doi: 10.3389/fnagi.2021.751375 (PMC8601448; doi:10.3389/fnagi.2021.751375)
Supplement: Supplementary file 1 [file Presentation_1.PPTX]

## Slide 1
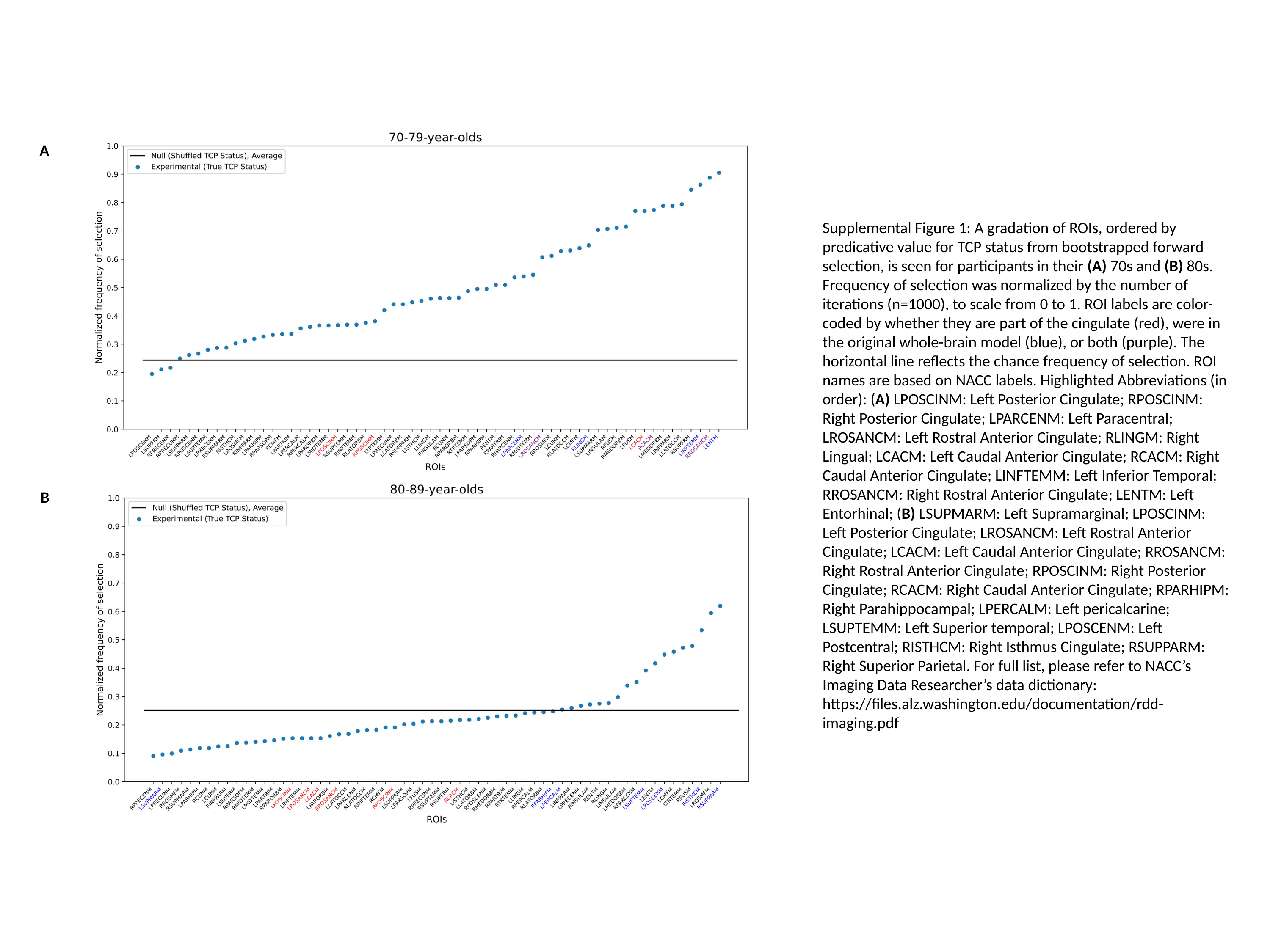

A
Supplemental Figure 1: A gradation of ROIs, ordered by predicative value for TCP status from bootstrapped forward selection, is seen for participants in their (A) 70s and (B) 80s. Frequency of selection was normalized by the number of iterations (n=1000), to scale from 0 to 1. ROI labels are color-coded by whether they are part of the cingulate (red), were in the original whole-brain model (blue), or both (purple). The horizontal line reflects the chance frequency of selection. ROI names are based on NACC labels. Highlighted Abbreviations (in order): (A) LPOSCINM: Left Posterior Cingulate; RPOSCINM: Right Posterior Cingulate; LPARCENM: Left Paracentral; LROSANCM: Left Rostral Anterior Cingulate; RLINGM: Right Lingual; LCACM: Left Caudal Anterior Cingulate; RCACM: Right Caudal Anterior Cingulate; LINFTEMM: Left Inferior Temporal; RROSANCM: Right Rostral Anterior Cingulate; LENTM: Left Entorhinal; (B) LSUPMARM: Left Supramarginal; LPOSCINM: Left Posterior Cingulate; LROSANCM: Left Rostral Anterior Cingulate; LCACM: Left Caudal Anterior Cingulate; RROSANCM: Right Rostral Anterior Cingulate; RPOSCINM: Right Posterior Cingulate; RCACM: Right Caudal Anterior Cingulate; RPARHIPM: Right Parahippocampal; LPERCALM: Left pericalcarine; LSUPTEMM: Left Superior temporal; LPOSCENM: Left Postcentral; RISTHCM: Right Isthmus Cingulate; RSUPPARM: Right Superior Parietal. For full list, please refer to NACC’s Imaging Data Researcher’s data dictionary: https://files.alz.washington.edu/documentation/rdd-imaging.pdf
B

## Slide 2
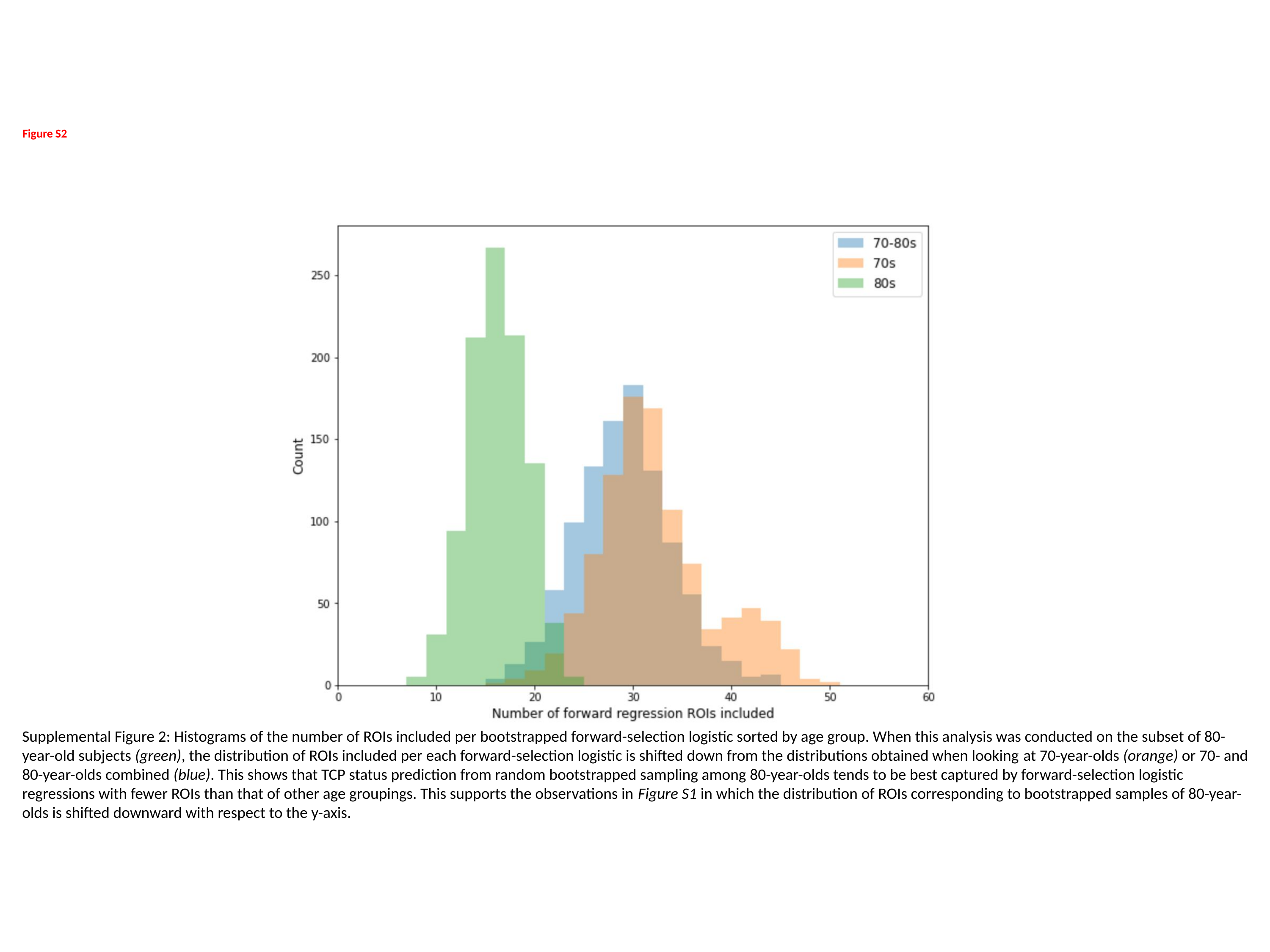

Figure S2
Supplemental Figure 2: Histograms of the number of ROIs included per bootstrapped forward-selection logistic sorted by age group. When this analysis was conducted on the subset of 80-year-old subjects (green), the distribution of ROIs included per each forward-selection logistic is shifted down from the distributions obtained when looking at 70-year-olds (orange) or 70- and 80-year-olds combined (blue). This shows that TCP status prediction from random bootstrapped sampling among 80-year-olds tends to be best captured by forward-selection logistic regressions with fewer ROIs than that of other age groupings. This supports the observations in Figure S1 in which the distribution of ROIs corresponding to bootstrapped samples of 80-year-olds is shifted downward with respect to the y-axis.

## Slide 3
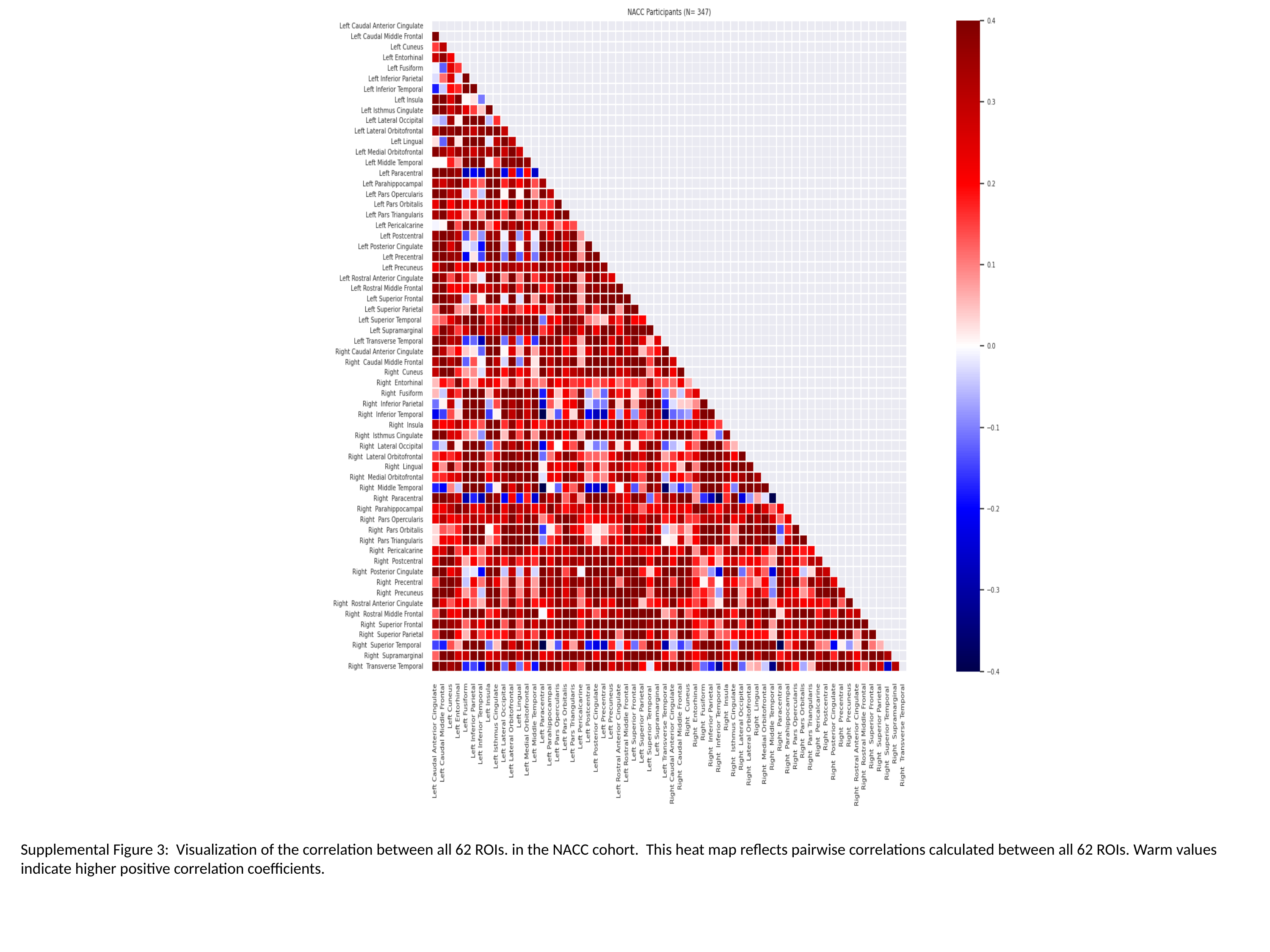

Supplemental Figure 3: Visualization of the correlation between all 62 ROIs. in the NACC cohort. This heat map reflects pairwise correlations calculated between all 62 ROIs. Warm values indicate higher positive correlation coefficients.

## Slide 4
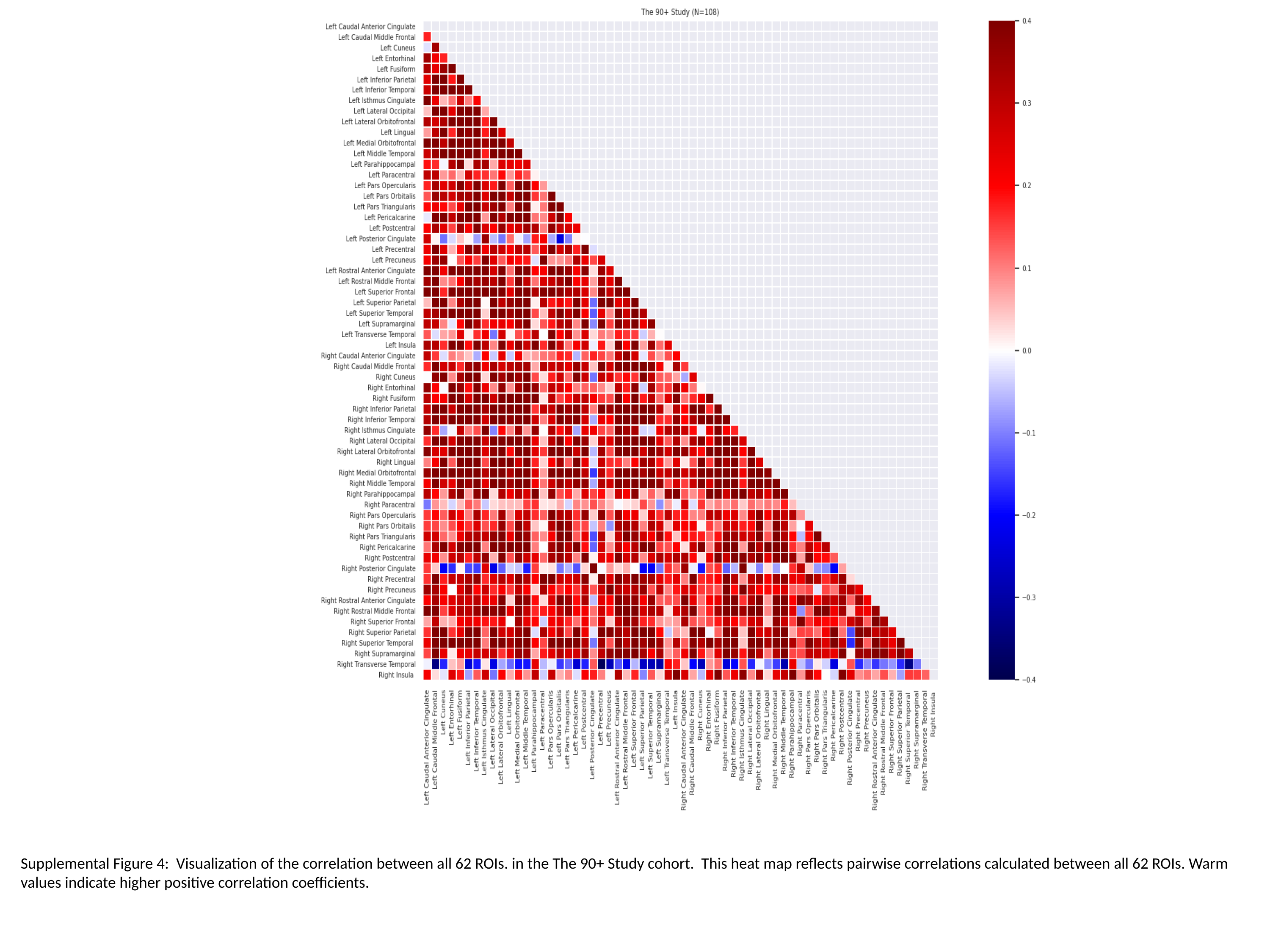

Supplemental Figure 4: Visualization of the correlation between all 62 ROIs. in the The 90+ Study cohort. This heat map reflects pairwise correlations calculated between all 62 ROIs. Warm values indicate higher positive correlation coefficients.
